# Determinants of desire for more children among women in Ethiopia

**DOI:** 10.1186/s12905-021-01563-3

**Published:** 2021-12-09

**Authors:** Mitiku Wale Muluneh, Yikeber Abebaw Moyehodie

**Affiliations:** Department of Statistics, College of Natural and Computational Science, Debre Tabor University, Debre Tabor, Ethiopia

**Keywords:** Desire, Children, Women, Logistic regression, Ethiopia

## Abstract

**Background:**

Desire for more children has an impact on couple’s fertility behaviors. It can be a precursor of actual fertility performance. However, the desire for more children is declining over time in Ethiopia. Therefore, this study aimed to identifying the determinants of the desire for more children among women in Ethiopia.

**Methods:**

The 2016 Ethiopian Demographic and Health Survey data were used for the analysis. The sample consisted of 15,683 women. The binary logistic regression model was used to assess the determinants of desire for more children among women in Ethiopia. The results are presented as crude odds ratios (COR) and adjusted odds ratios (AOR) together with their corresponding 95% confidence intervals.

**Results:**

No education (having no formal education) (AOR = 1.85, 95% CI 1.61–2.13), attained primary education (AOR = 1.62, 95% CI 1.43–1.83), age at first marriage 10–19 years (AOR = 1.80, 95% CI 1.27–2.54), Orthodox religion (AOR = 1.48, 95% CI 1.01–2.19), Catholic religion (AOR = 2.15, 95% CI 1.17–3.97), Muslim religion (AOR = 1.70, 95% CI 1.15–2.50), living in Amhara (AOR = 1.45, 95% CI 1.18–1.78), Oromia (AOR = 2.10, 95% CI 1.73–2.54), Benishangul (AOR = 1.17, 95% CI 1.01–1.45), SNNPR (AOR = 1.30, 95% CI 1.05–1.60), Gambela (AOR = 1.25, 95% CI 1.02–1.57), Harari (AOR = 2.24, 95% CI 1.82–2.76), ideal number of children four or fewer (AOR = 0.47, 95% CI 0.42–0.53), number of living children four or fewer (AOR = 2.12, 95% CI 1.90–2.37), and not use of contraceptives (AOR = 1.51, 95% CI 1.35–1.68) were associated with a higher desire for more children.

**Conclusion:**

This finding showed that the age of women, educational level, age at first marriage, religion, region, occupation, ideal number of children, number of living children, and use of contraceptives were significant determinants of desire for more children. Therefore, it is important to adopt programs to encourage the desire for more children, implement policies in an attempt to increase the total fertility rate in Ethiopia ought to critically consider these factors. Moreover, continuous education and knowledge on reproductive health will help for better fertility behaviour for the women.

## Background

Desire for more children has an impact on couple’s fertility behaviors. It can be a precursor of actual fertility performance. [1, 2]. The total fertility rate (TFR) is declining in the developed and developing countries. The TFR declined from 4.97 children per woman in 1950–1954 to 2.53 in 2005–2010 in the developing countries, and the changes were even more dramatic over this period, from a TFR of 6.08 to 2.69 [3]. All women have the right to produce their children. Women also have equal access to health services, when, and who they marry, and decide to desire more children. However, limiting births has a greater impact on fertility rates than birth interval and is a major factor driving fertility transition. Family planning programs prepare to meet demand by addressing supply and demand-side barriers to use [4].

Globally, in 1965 the average number of children per woman in the world had more than 5 children. However, the average number of children per woman is now below 2.5 children. The number of children per woman was halved. For more couples, the greatest source of anguish is that they have fewer children [5]. People have been producing fewer and fewer children every day compared to the last 50 years and have contributed to reducing the population sizes in many countries. In some countries in sub-Saharan Africa, fertility declines over time, especially in Ethiopia [6].

In Ethiopia, in 1992 the average fertility rate was 5.5 per women reproductive age. However, the average fertility rate was fewer than 2.3 children per reproductive age women in 2016, which is less than the required replacement fertility level [7–9]. This indicates that women’s desire for more children has been reduced by more than half. A declining population is a cause of reductions in economic activity, productivity, and economic security [10].

Various factors influence human fertility. These are proximate (direct) and distal (indirect) factors. Proximal factors are bio-behavioral factors known to be the intermediate determinants that are related to biological, reproductive, and behavioral/attitude factors through which the indirect determinants must directly affect fertility. Distal determinants, on the other hand, are socio-cultural factors that consist of socio-economic, and demographic factors that affect fertility indirectly by affecting bio-behavioral constraints. In addition, the fertility transition in Ethiopia shows that although family planning programs have been effective in accelerating the decline in fertility rates, fertility behaviors should be considered in the analysis of fertility trends [11]. Therefore, this study aimed to identify the determinants of the desire for more children among women in Ethiopia using Ethiopian Demographic and Health Survey (EDHS) 2016 data.

## Methods

### Study setting and design

This study was carried out in Ethiopia using the EDHS 2016 data to assess the determinants of the desire for more children among women. Ethiopia is located in the Horn of Africa (3°–14° N and 33°–48° E). The country covers 1.1 million square km, and it has a high central plateau that varies from 4550 m above sea level down to the Afar depression to 110 m below sea level. Administratively, Ethiopia is federally decentralized into nine regions, and two city administrations. The regions are divided into 68 zones with 817 administrative units called districts. The Ethiopian Demographic and Health Survey 2016 was a recent population-based cross-sectional survey conducted across the country [12].

### Data source and population

Secondary data were used in this study. This study was based on the 2016 Ethiopian Demographic and Health Survey (EDHS) data. The EDHS is a nationally representative survey conducted every five years in Ethiopia. The EDHS is a survey designed to provide population and health indicators at the national and regional levels. It was collected every five years. For this study, we used the fourth EDHS 2016, the recent EDHS. A stratified two-stage cluster sampling procedure was used in the survey. In the first stage, a total of 645 enumeration areas (202 in urban areas) were selected using systematic sampling with probability proportional to size. In the second stage, a fixed number of 28 households per cluster were randomly selected from the household listing [12]. The study population for this study included all women aged 15–49 years across all regions of Ethiopia. A total of 15,683 women of reproductive age were included in the final analysis.

### Variables of study

#### Outcome variable

The desire for more children was the outcome variable. The outcome variable was the dichotomous outcome, namely “want a (another) child,” coded as 1 and “want no more,” coded as 0. Hence, women who responded that they wanted another child were considered to have a desire for more children while those who responded that they wanted no more were considered as not having a desire for more children [10].

#### Independent variables

The study used eleven independent variables, these independent variables including age, residence, educational level, age at first marriage, religion, region, wealth index, occupation, ideal number of children, number of living children and use of contraceptives. These variables were considered because of their statistically significant relationships with desire for more children in previous studies [1, 11, 13]. Details of how each of these variables were coded can be found in Table 1.

**Table 1:** Result of women characteristics and desire for more children among women

Variable	Category	Desire for more children	
		Yes n = 10,274 (%)	No n = 5409 (%)
Age	15–24	5306 (82.9)	1095 (17.1)
	25–34	3579 (70.4)	1507 (29.6)
	35–49	1389 (31.1)	2807 (66.9)
Place of residence	Urban	3831 (71.6)	1517 (28.4)
	Rural	6443 (62.3)	3892 (37.7)
Educational level	No education	3898 (55.4)	3135 (44.6)
	Primary	3653 (70.1)	1560 (29.9)
	Secondary and above	2723 (79.2)	714 (20.8)
Age at first marriage	Oct-19	7592 (63.9)	4288 (36.1)
	20–29	2587 (71.1)	1054 (28.9)
	30–49	95 (58.6)	67 (41.4)
Religion	Orthodox	4202 (65.5)	2211 (34.5)
	Catholic	51 (56.0)	40 (44.0)
	Muslim	1676 (59.6)	1138 (40.4)
	Protestant	4243 (68.3)	1966 (31.7)
	Other	102 (65.4)	54 (34.6)
Region	Tigray	1174 (69.8)	508 (30.2)
	Afar	850 (75.4)	278 (24.6)
	Amhara	978 (56.9)	741 (43.1)
	Oromia	972 (51.4)	920 (48.6)
	Somali	1109 (79.7)	282 (20.8)
	Benishangul	700 (62.2)	426 (37.8)
	SNNPR	1120 (60.6)	729 (39.4)
	Gambela	662 (64.0)	373 (36.0)
	Harari	509 (56.2)	397 (43.8)
	Addis Ababa	1381 (75.7)	443 (24.3)
	Dire Dawa	819 (72.4)	312 (27.6)
Wealth index	Poor	4396 (65.4)	2322 (34.6)
	Middle	1857 (65.2)	991 (34.8)
	Rich	4021 (65.7)	2096 (34.3)
Occupation	No working	5492 (68.3)	2553 (31.7)
	Managerial	366 (74.1)	128 (25.9)
	Clerical	159 (75.4)	52 (24.6)
	Sales	1646 (66.0)	849 (34.0)
	Agricultural	1412 (53.5)	1225 (46.5)
	Household manual	1199 (66.6)	602 (33.4)
Ideal number of children	≤ 4	5340 (66.8)	2657 (32.2)
	> 4	4934 (64.2)	2752 (35.8)
Number of living children	≤ 4	9115 (73.2)	3337 (26.8)
	> 4	1159 (35.9)	2072 (64.1)
Use of contraceptives	No	8279 (66.9)	4092 (33.1)
	Yes	1995 (60.2)	1317 (39.8)

### Methods of data analysis

Data were extracted using the SPSS version 21 software and then exported to R version 4.0.3 statistical software for further analysis. Descriptive statistics including frequencies and percentages were computed to describe the study participants. A binary logistic regression model was used to identify factors associated with desire for more children. From binary logistic regression, we carried out a bivariate logistic regression analysis to examine the association between the independent variables and desire for more children. The results were presented using crude odds ratios (COR). Finally, we fitted a multivariable logistic regression model to examine the independent variables of desire for more children. The results were presented as adjusted odds ratio (AOR) together with their corresponding 95% confidence intervals signifying the level of precision. Multicollinearity was tested using the variance-inflation factor (VIF) test, suggesting that there was no multicollinearity since all variables had VIF < 5. To determine the goodness of fit is through the Homer-Lemeshow statistics, which is computed on data after the observations have been segmented into groups based on having similar predicted probabilities. Therefore, in this study, the Homer-Lemeshow test was greater than 0.05 (*P* value > 0.05), indicating that the model was a good fit [14].

## Results

A total of 15,683 women were included in the study, of these, 10,274 (65.5%) of women in Ethiopia desire for more children. The mean age (± standard deviation (SD)) of women in 2016 EDHS was 27.94 ± 9.16 years. Out of the total women, 7033 (44.8%) of women did not attend formal education and 3437 (21.9%) of the women attained secondary education and above. Almost 41% of these women were orthodox religion. Out of the total women, 12.1% of women were from Oromia region. 82.9% of women in the age group 15–24 years had a desire for more children. 71.6% of women living in urban areas had a desire for more children. Approximately 70% of the participants were from Tigray, 75.5% participants were from Afar and 56.9% of participants from Amhara regions had a desire for more children. 37.7% of women living in rural areas had no desire for more children (Table 1).

All independent variables were tested for crude association with desire for more children using bivariable binary logistic regression. In multivariable binary logistic regression analysis, variables including age, educational level, age at first marriage, religion, region, occupation, ideal number of children, number of living children, and use of contraceptives were significantly associated with the desire for more children among women in Ethiopia.

The odds of desire for more children among women with 15–24 years age group and 25–34 years age group was 0.13 (AOR = 0.13, 95% CI 0.11–0.14) and 0.23 (AOR = 0.23, 95% CI 0.20–0.25), respectively times lower compared to women who 35–49 years age group. The odds of desire for more children among women with no education and primary education were 1.85 (AOR = 1.85, 95% CI 1.61–2.13) and 1.62 (AOR = 1.62, 95% CI 1.43–1.83), respectively times higher compared to women who completed secondary and above education. The odds of desire for more children among women 10–19 years of age at first marriage were 1.80 times higher compared to those women 30–49 years of age at first marriage (AOR = 1.80, 95% CI 1.27–2.54). The odds of desire for more children among women who had Orthodox, Catholic, and Muslim religion were 1.48 (AOR = 1.48, 95% CI 1.01–2.19), 2.15 (AOR = 2.15, 95% CI 1.17–3.97), and 1.70 (AOR = 1.70, 95% CI 1.15–2.50), respectively times higher compared to those women who had other religion. The odds of desire for more children among women from Afar 0.68 (AOR = 0.68, 95% CI 0.54- 0.85), Somali 0.49 (AOR = 0.49, 95% CI 0.39–0.61) and Addis Ababa 0.83 (AOR = 0.83, 95% CI 0.72–0.97) regions were less likely as compared to women from Dire Dawa region. The odds of desire for more children among women from Amhara 1.45 (AOR = 1.45, 95% CI 1.18–1.78), Oromia 2.10 (AOR = 2.10, 95% CI 1.73–2.54), Benishangul 1.17 (AOR = 1.17, 95% CI 1.01–1.45), SNNPR 1.30 (AOR = 1.30, 95% CI 1.05–1.60), Gambela 1.25 (AOR = 1.25, 95% CI 1.02–1.57) and Harari 2.24 (AOR = 2.24, 95% CI 1.82–2.76) regions were more likely compared to women from Dire Dawa region. The odds of desire for more children among women who have not working status were 0.94 (AOR = 0.94, 95% CI 0.82–0.99) times lower compared to those women who have household manual status. The odds of desire for more children among women whose ideal number of children four or fewer were 0.47 (AOR = 0.47, 95% CI 0.42–0.53) times lower compared to women whose ideal number of children greater than four. The odds of desire for more children among women whose number of living children four or fewer were 2.12 (AOR = 2.12, 95% CI 1.90–2.37) times higher compared to women whose number of living children greater than four. The odds of desire for more children among women who do not use contraceptives were 1.51 (AOR = 1.51, 95% CI 1.35–1.68) times higher compared to those women who used contraceptives (Table 2).

**Table 2:** Factors Associated with Desire for more Children Among Women in Ethiopia using EDHS 2016 data

Variable	Category	COR 95% CI	AOR 95% CI
Age	15–24	0.10 (0.09–0.11)*	0.13 (0.11–0.14)*
	25–34	0.21 (0.19–0.23)*	0.23 (0.20–0.25)*
	35–49	1	1
Place of residence	Urban	0.66 (0.61–0.70)*	1.07 (0.95–1.20)
	Rural	1	1
Educational level	No education	3.07 (2.79–3.37)*	1.85 (1.61–2.13)*
	Primary	1.63 (1.47–1.80)*	1.62 (1.43–1.83)*
	Secondary and above	1	1
Age at first marriage	Oct-19	0.80 (0.58–1.10)	1.80 (1.27–2.54)*
	20–29	0.58 (0.42–0.80)*	1.38 (0.97–1.96)
	30–49	1	1
Religion	Orthodox	0.99 (0.71–1.39)	1.48 (1.01–2.19)*
	Catholic	1.48 (0.87–2.52)	2.15 (1.17–3.97)*
	Muslim	1.28 (0.91–1.80)	1.70 (1.15–2.50)*
	Protestant	0.88 (0.63–1.22)	1.33 (0.90–1.96)
	Other	1	1
Region	Tigray	1.14 (0.96–1.34)	0.82 (0.66–1.02)
	Afar	0.86 (0.71–1.04)	0.68 (0.54–0.85)*
	Amhara	1.99 (1.69–2.34)*	1.45 (1.18–1.78)*
	Oromia	2.49 (2.12–2.91)*	2.10 (1.73–2.54)*
	Somali	0.67 (0.56–0.80)*	0.49 (0.39–0.61)*
	Benishangul	1.60 (1.34–1.91)*	1.17 (1.01–1.45)*
	SNNPR	1.71 (1.46–2.01)*	1.30 (1.05–1.60)*
	Gambela	1.48 (1.23–1.78)*	1.25 (1.02–1.57)*
	Harari	2.05 (1.70–2.46)*	2.24 (1.82–2.76)*
	Addis Ababa	0.84 (0.71–0.99)*	0.83 (0.72–0.97)*
	Dire Dawa	1	1
Wealth index	Poor	1.01 (0.94–1.09)	1.08 (0.98–1.20)
	Middle	1.02 (0.93–1.12)	0.96 (0.86–1.07)
	Rich	1	1
Occupation	No working	0.92 (0.80–0.97)*	0.94 (0.82–0.99)*
	Managerial	0.70 (0.56–0.87)*	0.99 (0.76–1.28)
	Clerical	0.65 (0.47–0.90)*	0.88 (0.60–1.27)
	Sales	1.03 (0.90–1.17)	0.99 (0.87–1.15)
	Agricultural	1.73 (1.53–1.96)*	1.19 (1.02–1.38)*
	Household manual	1	1
Ideal number of children	≤ 4	0.89 (0.84–0.95)*	0.47 (0.42–0.53)*
	> 4	1	1
Number of living children	≤ 4	4.88 (4.50–5.30)*	2.12 (1.90–2.37)*
	> 4	1	1
Use of contraceptives	No	1.62 (1.45–1.81)*	1.51 (1.35–1.68)*
	Yes	1	1

1 shows reference category

*shows significant at 0.05 level of significance

## Discussion

The purpose of this study was to identify the determinants of desire for more children among women in Ethiopia. A sample of 15,683 women were included from the 2016 EDHS data. The study found out that 65.5% of women desired more children. The odds of desire for more children among women 15–24 and 25–34 years age group was lower as compared to those women 35–49 years age group. The finding is in contrast with the other studies [15]. Due to the increasing age of women in Ethiopia, it is necessary to develop a childbearing culture at the right time for couples, and couples are worried about childbearing at old ages.

Furthermore, the study revealed that having no education was positively associated with desire for more children at the multivariate analysis, suggesting that no education was associated with higher desire for more children compared to secondary and above education level. This finding is in line with the previous findings on this subject that higher education is associated with lower fertility desire [16]. The possible explanation for this result may be that no educated women have enough time for childbearing. Furthermore, they indicated that education improves the ability of women to implement simple health knowledge and facilitates family planning methods and contraception use.

This study showed that women’s age at first marriage influenced desire for more children. Women in the age 15–24 years were more likely to desire for more children compared to those women whose age group 30–49 years. Desire for more children lowered when age at first marriage of the women increased. This finding is in line with previous studies that higher socio-demographic factors were associated with a lower desire for more children [1, 17]. This might be that early marriage is deeply rooted in religious and cultural traditions of Ethiopian communities and this usually results in the early marriage of children without their consent and letting them decide on their own.

In addition, the study showed that Muslim and Catholic religious women were more likely to desire for more children than other religious women. It could be that religion has an immense social, economic, and political significance in most societies, and thus plays an important role in sanctioning or promoting acceptance or creating resistance to family planning. This finding is in line with a study done in Nepal [18]. The study showed that Orthodox religious women were more likely to desire more children compared to other religious women. However, it is contradicted with the finding of other studies in Kenya, and Tanzania that have shown that Christian women have less desire for more children than other religious women [19]. The probable explanation religious tradition strongly influences the uptake of family planning, with a wide range of interpretations of most religious traditions (religious educations) affecting the perceived acceptability of government family planning programs.

The odds of desire for more children among women in Amhara, Oromia, Benishangul, SNNPR, Gambela and Harari regions were higher than women in the Dire Dawa region. This finding is in line with the other studies in Ethiopia [12]. However, the odds of desire for more children among women in Afar, Somali, and Addis Ababa were lower than women in the Dire Dawa region. This finding is consistent with the results of EDHS 2016 [12]. This might be due to culture, urbanization, and religious differences, as well as disparities in the implementation of desire for more children actions across different regions of Ethiopia.

The study showed that women with four or fewer ideal numbers of children were less likely to desire for more children. Women with four or fewer living children may be satisfied with their ideal number of children. The study shows that women with four or fewer living children were more likely to desire more children. This study was consistent with the other study. Women with four or fewer living children may be dissatisfied with their existing number of children. The study revealed that women who use contraceptives were less likely to desire for more children. This finding was consistent with previous studies [20–22]. The probable explanation is that women who are using contraceptives might not wish to give birth to additional children.

This study aimed to analyze the determinant factors on desire for more children, an indicator of behavior fertility in Ethiopia. To the best knowledge of researchers, few studies investigated desire for more children and the effects of different variables on that. It is worth mentioning that can be applied in future studies since the results of the current study were obtained based on a large representative sample and analyzed a lot of potential factors that determine desire for more children. The finding of this study can pave the way for policymakers to design and adopt fertility planning programs.

### Strength and limitation of the study

The data were collected using standard and validated data collection tools, relatively large sample size, and nationally representative data. This helps to provide better representative data as all regions and administrative cities in the country. However, the limitation is the cross-sectional data does not show the cause-and-effect relationship between variables.

## Conclusion

The main goal of the current study was to identify the determinants of desire for more children among women in Ethiopia. This study showed that the age of women, educational level, age at first marriage, religion, region, occupation, ideal number of children, number of living children, and use of contraceptives were important factors significantly associated with the desire for more children. The study concludes that women with no education, primary education, 10–19 years age at first marriage, Orthodox religion, Catholic religion, Muslim religion, Amhara region, Oromia region, Benishangul region, SNNPR region, Gambela region, Harari region, agricultural occupation, number of living children four or fewer, and not use of contraceptives were positively associated to desire more children. However, 15–24 and 25–34 years of age groups, no working status, ideal number of children four or fewer, Somali and Afar region was negatively associated with desire for more children. Therefore, the finding is important to adopt programs to encourage the desire for more children and increase the fertility rate by considering these factors critically. Moreover, continuous education and knowledge on reproductive health will help for better fertility behaviour for the women.

## Data Availability

The data is available at https://www.dhsprogram.com/data/dataset_admin/login_main.cfm for this study.

## Abbreviations

AOR: Adjusted Odds Ratio
CI: Confidence Interval
COR: Crude Odds Ratio
EDHS: Ethiopian Demographic and Health Survey
IRB: Institutional Review Board
SNNPR: Southern Nations, Nationalities and Peoples' Region
TFR: Total Fertility Rate
